# Dr. W. G. Grace

**Published:** 1916-07

**Authors:** 


### DR. W. G. GRACE.

Of William Gilbert Grace, whose death took place in October last, much has been written during his life, and more would have been written at his death if it had occurred at any other time than the present. He was born July 18th, 1848, entered the Medical School in 1868, and qualified M.R.C.S. and L.R.C.P. in 1879, taking rather a long time between his "primary" and "pass." The writer of this memoir well remembers sitting next to the great man at the examination at the College of Surgeons, when both successfully went in for their primary.

W. G. settled in Stapleton Road, and was Medical Officer to No. 2 District Barton Regis Union from 1879 to 1898, also Medical Examiner for the Prudential Society and during the time that he lived in Bristol and afterwards in Victoria Square,

Clifton, did a considerable amount of medical work, especially in the winter months.

One incident may be mentioned to show that W. G. was not merely a cricketer. When the writer was Surgeon for the week at the Bristol Royal Infirmary five men were admitted one morning all badly burnt in an explosion at a colliery to which W. G. was medical officer. They asked for him, and he called in to see how they were. Although they were much covered by wool dressings, when he came he knew them all by name, and his words of sympathy comforted them not a little.

His cricket career has been treated of elsewhere; suffice it to say that his pre-eminence has never been challenged. He left Clifton in 1899 and lived at Sydenham. Here he played some cricket, and was often seen running with the beagles; and he played a good deal of bowls and was very fond of shooting, being a good shot. After giving up more active sports he went to reside near Eltham, where his garden gave him the greatest enjoyment.

Coming before the public eye as much as he did during at least thirty-five years of his life, through all the summer every school-boy and nearly everybody else looking into the papers to see what W. G. had done, it is remarkable that he was such an unaffected and simple-minded man. In private life he was a most affectionate husband, father, and friend, and he leaves a void that none can fill.

He lost his only daughter, Bessie, when she was twenty years old, just as he was leaving Clifton, and a few years later young W. G., a master at the Royal Naval College at Osborne, and ex-captain of the Clifton College Eleven, died of appendicitis; and there are left his widow and two sons, Edgar, Captain of H.M.S. Grafton (he was at the Dardanelles at the time of his father's death, and he himself has a son a midshipman just going to sea), and C. B. Grace, Major in the Kent Fortress Engineers.

